# Sensitivity analysis of point neuron model simulations implemented on neuromorphic hardware

**DOI:** 10.3389/fnins.2023.1198282

**Published:** 2023-08-24

**Authors:** Srijanie Dey, Alexander G. Dimitrov

**Affiliations:** Department of Mathematics, Washington State University, Vancouver, WA, United States

**Keywords:** neuromorphic computing, LIF models, neural simulations, validation, sensitivity analysis

## Abstract

With the ongoing growth in the field of neuro-inspired computing, newly arriving computational architectures demand extensive validation and testing against existing benchmarks to establish their competence and value. In our work, we break down the validation step into two parts—(1) establishing a methodological and numerical groundwork to establish a comparison between neuromorphic and conventional platforms and, (2) performing a sensitivity analysis on the obtained model regime to assess its robustness. We study the neuronal dynamics based on the Leaky Integrate and Fire (LIF) model, which is built upon data from the mouse visual cortex spanning a set of anatomical and physiological constraints. Intel Corp.'s first neuromorphic chip “Loihi” serves as our neuromorphic platform and results on it are validated against the classical simulations. After setting up a model that allows a seamless mapping between the Loihi and the classical simulations, we find that Loihi replicates classical simulations very efficiently with high precision. This model is then subjected to the second phase of validation, through sensitivity analysis, by assessing the impact on the cost function as values of the significant model parameters are varied. The work is done in two steps—(1) assessing the impact while changing one parameter at a time, (2) assessing the impact while changing two parameters at a time. We observe that the model is quite robust for majority of the parameters with slight change in the cost function. We also identify a subset of the model parameters changes which make the model more sensitive and thus, need to be defined more precisely.

## 1. Introduction

Design that is inspired by the structure and characteristics of neurons and the complex networks formed by them, is called neuromorphic. A neuromorphic system, as the name suggests—“like the brain” can mimic the brain's function in a truer sense as their design is analogous to the brain (Thakur et al., 2018; Roy et al., 2019). The term neuromorphic was originally conceived by Mead (1989) during the 1980s to define very large scale integration (VLSI) systems that contained electronic circuits based on the biological design of the nervous system. At present however, the term “neuromorphic” encompasses broader ideas such as computing systems that contain digital processors that are capable of simulating neural models of computations, and also artificial learning algorithms and machine learning techniques that emulate biologically relevant neural networks and learning mechanisms (Bhuiyan et al., 2010; Sharp and Furber, 2013; Davies et al., 2018).

It is worth emphasizing that, although neuromorphic systems hold the power to revolutionize the modern computing system, rigorous validation and benchmarking remains to be done to a great extent in order to be able to fully utilize their capabilities. Building efficient tools to map algorithms to these architectures, estimating their performance along with creating a suite of benchmarks for different domains of applications queue up as some of the initial challenges in using these neuromorphic systems. Thus, in our work we aim to establish a framework to be able implement the classical simulations on Loihi and investigate the feasibility of the execution using different validation techniques.

Taking advantage of technology combined with scalable architectures, neuromorphic capabilities allow building models with millions of neurons and billions of synapses. Thus, based on this potential we work on developing a principled approach toward obtaining simulations of biologically relevant neural network models on a novel neuromorphic hardware platform. The challenge arises from the difference in hardware and software paradigms in use for the neuromorphic systems. This manuscript focuses on establishing a platform to enable these implementations along with investigating the trade-offs necessitated by these simulations.

For our work, we focus on the Loihi architecture (Davies et al., 2018), as at present it is one of the most powerful platforms with specialized digital hardware and significant software support. While the earlier TrueNorth (DeBole et al., 2019) has a similar combination of hardware and programming support, its inter-neuron connectivity capability is relatively limited; Loihi approaches the human-scale connectivity density of interest to our research. SpiNNaker has similar capabilities, but is constructed of standard CPU hardware (Khan et al., 2008). Loihi's capabilities on the other hand, are built-in on a chip, thus forcing us to explore new programming paradigms. And recent and current state of the art hybrid analog-digital platforms, like Neurogrid (Benjamin et al., 2014), Braindrop (Neckar et al., 2019), DYNAP-SE2 (Moradi et al., 2018), and BrainScaleS(2) (Pehle et al., 2022) are beyond the scope of this manuscript. However, we believe that the simulation and programming paradigms developed on the Loihi platform can generalize to these analog platforms as well, and thus decrease the development time on these unfamiliar architectures.

In Dey and Dimitrov (2022) we presented initial results in mapping classical neuronal models to the Loihi architecture, quantifying discrepancies due to the differences between classical and neuromorphic numeric constraints. Despite all differences, simulated states were shown to be correlated within 1 − 10^−5^, showing that the Loihi architecture can offer good support for neural simulation projects. The next step in the validation process is to check how robust the simulations are to variations in the model parameters. We want to examine how perturbing the parameters in the allowed range changes the validation results. Since the implementation in both systems follow different hardware and programming paradigms, it becomes pertinent to check if changing the variables impact the simulation results and hence the validation criteria.

This manuscript lays down the groundwork for parameter perturbation and sensitivity analysis between the model implementations in the two platforms. We establish theorems that assess the error trend related to the parameter variation and mathematically investigate the contribution of the parameters. In addition, we resolve model intricacies in order to be able to implement the perturbation and sensitivity techniques using simulations in both systems and cross-check the results in relation to the established theorems to analyze the robustness of the models. Finally, we summarize our findings and lay the ground for future work with expected improvements based on the second generation of the neuromorphic Loihi chip named Loihi 2 (Intel, 2022), which promises to deliver greater functionality, more programming flexibility, performance acceleration and smoother integration to larger systems.

### 1.1. Why validate?

Neuromorphic systems hold great potential with respect to new computation systems, focusing on improved efficiency and processing. They also have the potential to accelerate research in Neuroscience, as a medium to learn extensively about the brain. However, in order to exploit those possibilities, it becomes pertinent that this technology is systematically tested and validated against standard benchmarks that exist for today's computing systems.

In order to execute this validation, a platform for comparison between the neuromorphic and the classical/conventional systems needs to be established. Thus, the first step in the process is to be able to implement standard simulations in the neuromorphic systems. However, this step is significant and non-trivial because the programming paradigm and the architecture of neuromorphic systems is significantly different from the conventional computing systems.

To highlight one of the main differences in computing architectures, the fundamental computing element of a neuromorphic system is the spiking neural networks (SNNs), which is an artificial neural networks inspired by biology. Specifically, an SNN consists of interconnected neurons *with intrinsic dynamics* (stateful neurons). These neurons communicate with each other using spikes, via local memory elements (synapses), similar to how a biological neural network operates in the brain. In principle the neuromorphic units are also analog—however, unlike digital computers, they use subthreshold dynamics of electronic elements. This is very different from how present computing systems process information (Brette et al., 2008). In particular, current computers use numbers as fundamental computing elements, either for logic, or to build dynamic simulations iteratively. The prevalent von Neumann/Harvard architecture also uses a shared memory model with local computational modules (CPU/ALU). Those are the main contrast of interest to us with neuromorphic systems, in which the lowest level of abstraction is already a dynamical system, with memory local to each dynamical element. That pits the almost infinite reconfigurability of classical computational architectures against the specialized, but very energy- and resource-efficient neuromorphic computing environments, with yet-unknown scope of computing power. Intel's Loihi, discussed here, can be considered as a transition technology in that direction. It provides stateful neural models with intrinsic, configurable dynamics, memory local to a small group of neurons, and simulates noise sources that are typical in analog systems. However, Loihi is a digital system which implements all that in the standard threshold mode of digital operation, with 0- and 1-s. This technology allows us to develop methods for configuration and analysis of neuromorphic systems with persistent architecture and tools, which can then be transferred more readily to analog neuromorphic systems, with their more challenging operating models. Thus, after addressing the differences once we obtain the neuromorphic simulations, we compare them to the simulations in the classical systems and assess how well the new simulations fare.

Here, we take the analysis of our implementations a step further by trying to assess the relationship between the models and their parameters. As these neural models rely on a combination of multiple parameters, it becomes necessary to investigate which model input affects the model output the most. Moreover, since we work with two very different platforms, comparing the uncertainty in the model outputs based on the various parameters gives us an additional point of view to the validity of the implementations. We do this using a method called perturbation and sensitivity analysis.

### 1.2. Summary of prior work

We provide a brief summary of the methods used in Dey and Dimitrov (2022), for reference in the subsequent work on sensitivity analysis.

#### 1.2.1. Problem formulation

A typical neuron consists of a soma, dendrites and a single axon. Neurons send signals along an axon to a dendrite through junctions called synapses. The classical Leaky Integrate and Fire (LIF) equation (Gerstner and Kistler, 2002) is a point neuron model which reduces much of the neural geometry and dynamics in order to achieve computational efficiency. Though these point neuron models are extensively simple, they can mimic realistic neuron activity for a variety of cell types (Izhikevich and Edelman, 2008; Yamauchi et al., 2011). Moreover, these simplified models can be efficiently evaluated numerically allowing systemic investigation as compared to the biophysical models, which are harder to analyze in the extensive parameter space and also incur huge computational cost. Earlier work in different neuromorphic platforms like SpiNNaker (Furber and Temple, 2007) and TrueNorth (Cassidy et al., 2013) have demonstrated successful modeling of large-scale neurons based on these point-neurons models and thus motivates our implementations.

The LIF model is one of the simplest and rather efficient representations of the dynamics of the neuron, while still providing reasonable approximation of biological neural dynamics (Lazar, 2007) for some classes of neurons (Teeter et al., 2018). It is stated mathematically as:


(1) $$\begin{array}{l} V^{\prime }\left(t\right)=\frac{1}{C}\left[I_{e}\left(t\right)-\frac{1}{R}\left(V\left(t\right)-E_{L}\right)\right] \end{array}$$

(2) $$\begin{array}{l} V\left(t\right)\leftarrow V_{r}\text{, if }V\left(t\right)>\text{Θ} \end{array}$$

where, *V*(*t*), membrane potential (state); *C*, membrane capacitance (parameter); *R*, membrane resistance (parameter); *E*_*L*_, resting potential (parameter); *I*_*e*_, trans-membrane current (control and state); *V*_*r*_, reset membrane potential; Θ, firing threshold.

As Loihi encapsulates the working of an SNN, it implements its computational model as a variation of the LIF model based on two internal state variables: the synaptic current and the membrane potential (Davies et al., 2018).


(3) $$\begin{array}{l} v^{\prime }\left(t\right)=-\frac{1}{\tau_{v}}v\left(t\right)+u\left(t\right) \end{array}$$

(4) $$\begin{array}{l} u\left(t\right)=\underset{j}{\sum }w_{j}\left(\alpha_{j}*\sigma_{j}\right)\left(t\right)+b \end{array}$$

(5) $$\begin{array}{l} v\left(t\right)\leftarrow 0\text{, if }v\left(t\right)>\theta \end{array}$$

where, *v*(*t*), membrane potential; *u*(*t*), synaptic current; *w*, synaptic weight; α, synaptic response function; *b*, constant bias current; τ_*v*_, time constant; θ, firing threshold.

#### 1.2.2. Classical simulation platforms

Next, we lay down the details of our simulation platforms. We begin our discussion with the classical platform. For implementing a simulation on this platform, we use the Brain Modeling Toolkit (BMTK) (Dai et al., 2020) developed by the AIBS. The data used for the simulations is based on brain database by AIBS which consists of a large amount of data based on electrophysiological, morphological, and transcriptomic measurements of individual cells from both human and mouse brain as well as a plethora of models simulating cell activity, thus providing us with a multi-level validation substrate, ranging from single cells to small ensembles and finally expanding to whole mouse ViSp-scale networks. Being open source, these resources enable us to experiment with a varied range of data and thus support our extensive validation of neuronal models in the neuromorphic system.

BMTK works with different simulation backends to facilitate these multiple model resolutions. It consists of a Builder module to create the models and four respective simulator modules, namely—BioNet, PointNet, PopNet, and FilterNet to simulate the models. BioNet works with NEURON as the backend to facilitate detailed Biophysical Models. PointNet supports the simulation of highly efficient Point Models with the help of NEST (Linssen et al., 2018). FilterNet provides an interface to BMTK's built-in solver of input-output transformations and finally PopNet allows simulations based on population-statistical models by interfacing with the DiPDE tool (Cain et al., 2016), which supports a population density approach for simulations of coupled networks of neuronal populations. In this study, we work with the Point Neuron Models with simulations supported by the BMTK module PointNet via NEST 2.11+ (Linssen et al., 2018). For analysis and visualization, we use the HDF5 output format.

#### 1.2.3. Mapping to Loihi dynamics

For our neuromorphic platform, Intel's fifth and most complex fabricated chip by 2018 “Loihi”^1^ provides us with the tools to implement and test out the various neuromorphic features. The output provided by Loihi simulations is then compared to the output of classical simulations implemented in BMTK.

To map piecewise-continuous differential equation models like (1) into the discrete, fixed-point implementation of Loihi, we rescale both state and time of the LIF model as


(6) $$\begin{array}{l} v=\left(V-V_{r}\right)/V_{s}. \end{array}$$

The *v*(*t*) state evolves on-chip according to the update rule,


(7) $$\begin{array}{l} v\left(t+1\right)=v\left(t\right)\left[1-\frac{\delta_{v}}{2^{12}}\right]+b+u\left(t\right) \end{array}$$

where δ_*v*_ is the membrane potential decay parameter and *b* is the constant bias current listed in Equation (4). We define the Loihi voltage decay parameter δ_*v*_ in terms of the timestep *dt*, as,


(8) $$\begin{array}{l} \left(2^{12}-\delta_{v}\right)\;2^{-12}=\left(1-\frac{dt}{\tau_{v}}\right) \end{array}$$

(9) $$\begin{array}{l} \;\Rightarrow \;\delta_{v}=\frac{dt}{\tau_{v}}\;2^{12}=\frac{dt}{RC}\;2^{12} \end{array}$$

Table 1 shows the external spike-times used in the simulations, which are generated by five spike sources using a random Poisson spike generator with a max firing rate of 5 Hz and then frozen to stimulate the different models in both BMTK and Loihi.

**Table 1:** External spike timestamps that serve as network stimulus.

**Source**	**Spike -Times (ms)**
0	446
1	355
2	53, 258, 300, 424, 457
3	88, 466
4	100, 212

## 2. Sensitivity analysis

We deal with our cost function *particularly* in terms of the BMTK and Loihi LIF models and examine the rate of change of the cost function based on the chosen parameters.

### 2.1. Derivative-based analysis

In this section, we restate our ℓ_2_-cost function specifically based on our LIF mathematical model and use derivative based analysis to mathematically establish the nature of contribution we expect to see from each of the parameters in the model.

As stated in Dey and Dimitrov (2022), the cost function Root Mean Squared Error (RMSE) is written as :


(10) $$\begin{array}{l} RMSE=\sqrt{\frac{1}{n}\overset{n}{\underset{i=1}{\sum }}{\left(y_{L}^{i}-y_{B}^{i}\right)}^{2}} \end{array}$$

where, *i*, index of data point; *y*_*L*_, transformed Loihi voltage trace; *y*_*B*_, original BMTK voltage trace; *n*, number of data points.

We aim to investigate the impact of three primary parameters, namely *E*_*L*_, τ_*v*_, and *C*_*m*_ from (1). The motivation for choosing the three parameters arises from the model definition and the results established in Dey and Dimitrov (2022). As we deal with a *fixed-sized discrete time-step* model, our previous analysis was based on two main control values—*V*_*s*_ and *dt*, which regulated the spatial and temporal scales of the simulations. Thus, based on this idea we choose to examine the contribution of the parameters that fall under these control values.

#### 2.1.1. Choosing the parameters

For *V*_*s*_, we choose *E*_*L*_ which is the resting potential and contributes directly toward the membrane potential *V*(*t*) (Equations 6, 1) and for *dt*, we choose τ_*v*_ and *C*_*m*_, as τ_*v*_ (Equation 8) is the membrane time constant and controls the evolution of the membrane dynamics via the decay parameter (and *C*_*m*_ = τ_*v*_/*R*).

The sensitivity analysis section gives us a theory based on the errors calculated for both *V*_*s*_ and *dt* that contribution by parameters under *V*_*s*_ is higher than those under *dt*. (To calculate the normalized errors for each of *V*_*s*_ and *dt*, we choose the difference of the lowest and highest errors and normalize it with the help of the difference in the respective precision scale. For *V*_*s*_, we find it to be ≈ 3.3333 and for *dt*, we have ≈ 0.00166).

Thus, keeping the notation similar as used in the previous section, we propose:

Theorem 2.1. Let $C\left(p_{j}\right):P\to \mathbb{R}$, $P\subseteq {\mathbb{R}}^{n}$, *j* = 1, 2, 3 be the above cost function, with *p*_1_, *p*_2_, *p*_3_ denoting the parameters *C*_*m*_, τ_*v*_, and *E*_*L*_ respectively, with *y*_*L*_ and *y*_*B*_ composed of parameters $p_{j}\in P$. Then, given $|C\left(p_{j}\right)|<1$, the cost function is most impacted by variation in *E*_*L*_.

*Proof*. We establish the proof with help of the partial derivative of $C\left(p_{j}\right)$ with respect to the parameters *p*_1_, *p*_2_, *p*_3_ which are *C*_*m*_, τ_*v*_, and *E*_*L*_ respectively.

The first step is to establish the RMSE in Equation (10) in terms of the parameters.

Based on the mapping reported in Dey and Dimitrov (2022),


(11) $$\begin{array}{l} y_{B}-y_{L}=V^{\prime }\left(t\right)-v^{\prime }\left(t\right) \end{array}$$

(12) $$\begin{array}{l} =V^{\prime }\left(t\right)-\left[-\frac{1}{\tau_{v}}v\left(t\right)+u\left(t\right)\right] \end{array}$$

where,


$$\begin{array}{l} v\left(t\right)=\frac{V\left(t\right)-V_{r}}{V_{s}},\\ u\left(t\right)=\frac{1}{CV_{s}}I_{e}\left(t\right)+\frac{1}{\tau_{v}}\frac{E_{L}-V_{r}}{V_{s}},\\ \;\tau_{v}=RC. \end{array}$$

Thus,


$$\begin{array}{l} y_{B}-y_{L}=-\frac{1}{\tau_{v}}V\left(t\right)+\frac{I_{e}\left(t\right)}{C}+\frac{E_{L}}{\tau_{v}}\\ \;+\frac{V\left(t\right)}{\tau_{v}V_{s}}-\frac{-V_{r}}{\tau_{v}V_{s}}-\frac{I_{e}\left(t\right)}{CV_{s}}-\frac{E_{L}-V_{r}}{\tau_{v}V_{s}}\\ \;=\frac{V\left(t\right)}{\tau_{v}}\left(\frac{1}{V_{s}}-1\right)+\frac{I_{e}\left(t\right)}{C}\left(1-\frac{1}{V_{s}}\right)+\frac{E_{L}}{\tau_{v}}\left(1-\frac{1}{V_{s}}\right) \end{array}$$

which makes,


$$\begin{array}{l} y_{B}-y_{L}=\left(\frac{1}{V_{s}-1}\right)\left[\frac{V\left(t\right)}{\tau_{v}}-\frac{I_{e}\left(t\right)}{C}-\frac{E_{L}}{\tau_{v}}\right]. \end{array}$$

This gives us,


$$\begin{array}{l} {\left(y_{B}-y_{L}\right)}^{2}=\frac{1}{{\left(V_{s}-1\right)}^{2}}{\left[\frac{V\left(t\right)-E_{L}}{\tau_{v}}-\frac{I_{e}\left(t\right)}{C}\right]}^{2}\\ =\frac{1}{{\left(V_{s}-1\right)}^{2}}\left[\frac{{\left(V\left(t\right)-E_{L}\right)}^{2}}{\tau_{v}^{2}}+\frac{I_{e}^{2}\left(t\right)}{C^{2}}-\frac{2\left(V\left(t\right)-E_{L}\right)I_{e}\left(t\right)}{\tau_{v}C}\right]. \end{array}$$

Finally, the RMSE stands as:


(13) $$\begin{array}{l} RMSE=\\ \frac{1}{\sqrt{n}\left(V_{s}-1\right)}\sqrt{\overset{n}{\underset{i=1}{\sum }}\left[\frac{{\left(V^{i}\left(t\right)-E_{L}\right)}^{2}}{\tau_{v}^{2}}+\frac{I_{e}^{2}\left(t\right)}{C^{2}}-\frac{2\left(V^{i}\left(t\right)-E_{L}\right)I_{e}\left(t\right)}{\tau_{v}C}\right]}. \end{array}$$

Thus, letting


$$\begin{array}{l} e=\left[\frac{{\left(V^{i}\left(t\right)-E_{L}\right)}^{2}}{\tau_{v}^{2}}+\frac{I_{e}^{2}\left(t\right)}{C^{2}}-\frac{2\left(V^{i}\left(t\right)-E_{L}\right)I_{e}\left(t\right)}{\tau_{v}C}\right] \end{array}$$

from Equation (13) and finding its partial derivative w.r.t *C*_*m*_, τ_*v*_, and *E*_*L*_ we get:


(14) $$\begin{array}{l} \frac{\partial \left(e\right)}{\partial C}=\left[\frac{-2I_{e}^{2}\left(t\right)}{C^{3}}+\frac{2\left(V^{i}\left(t\right)-E_{L}\right)I_{e}\left(t\right)}{\tau_{v}C^{2}}\right] \end{array}$$

(15) $$\begin{array}{l} \frac{\partial \left(e\right)}{\partial \tau_{v}}=\left[\frac{-2{\left(V^{i}\left(t\right)-E_{L}\right)}^{2}}{\tau_{v}^{3}}+\frac{2\left(V^{i}\left(t\right)-E_{L}\right)I_{e}\left(t\right)}{\tau_{v}^{2}C}\right] \end{array}$$

(16) $$\begin{array}{l} \frac{\partial \left(e\right)}{\partial E_{L}}=\left[\frac{-2\left(V^{i}\left(t\right)-E_{L}\right)}{\tau_{v}}+\frac{2I_{e}\left(t\right)}{\tau_{v}C}\right]. \end{array}$$

From Equation (16), we find that the partial derivative of the RMSE w.r.t *E*_*L*_ has a linear form whereas from Eqs. (14) and (15) we see the derivatives are non-linear. Since the RMSE values have been found to be always < 1 (Chapter 4) given by $|C\left(p_{j}\right)|<1$, we can infer that with a linear rate of change deviation from the minima will result in stark changes in RMSE as compared to a non-linear rate of change. Thus, we expect to find that *E*_*L*_ impacts the model more significantly as compared to *C*_*m*_ and τ_*v*_.

Proposition 1. Let $C\left(p_{j}\right):P\to \mathbb{R}$, $P\subseteq {\mathbb{R}}^{n}$, *j* = 1, 2, 3 be the above cost function, with *p*_1_, *p*_2_, *p*_3_ denoting the parameters *C*_*m*_, τ_*v*_, and *E*_*L*_ respectively, with *y*_*L*_ and *y*_*B*_ composed of parameters $p_{j}\in P$. Then, given $|C\left(p_{j}\right)|<1$:

1. *E*_*L*_ impacts the model more significantly as compared to *C*_*m*_ and τ_*v*_.

In the next section, we perform the perturbation and sensitivity analysis based on simulations in both BMTK and Loihi. In addition to enabling the simulations by addressing the modeling differences in both the platforms, we also verify the important results obtained in the sections above.

## 3. Perturbation and sensitivity simulation results

The goal of this section is to examine the contribution of the state parameters on the model dynamics via the state variables membrane potential and current with the help of simulations, i.e., we want to examine how changes in the parameters affect the results of proposed direct mapping between the two platforms (Dey and Dimitrov, 2022). This serves the purpose of elucidating the significance of each state parameter in the model, and identifying parameters for effective formal optimization procedures.

### 3.1. Implementing sensitivity analysis methods

For this study, we choose to work with a Local Sensitivity Analysis method—more specifically the OAT method. As our models are validated prior to performing the sensitivity analysis, our foremost aim is to check the credibility of the implementations based on each parameter variation. We assess the impact relative to the changes in the cost function value.

For our work we use the OAT method and one of its variations to perform sensitivity analysis on our models. We implement the sensitivity analysis for both the BMTK and Loihi simulations and then analyze them.

#### 3.1.1. One-at-a-time method

In the first method, we use the one-at-a-time (OAT) approach. As mentioned earlier, OAT involves:

- Analyzing the effect of one parameter on the cost function while keeping the other parameters intact.
- Returning the parameter to its original value, then repeating the method for the other parameters.

Since we use the RMSE as the cost function for the analysis, we monitor the sensitivity of the model response by assessing the changes in the output in terms of the RMSE.

It is to be noted here that we are working on two models—the BMTK model and the Loihi model. Thus, to perform the analysis, we approach the problem from two directions. First, we keep the parameters in the Loihi model fixed/original and alter the parameters in the BMTK model one at a time. The next step is to analyze how modifying the parameter affects the results in terms of the RMSE. We work on a range of parameters during each run. The new RMSE looks as follows:


(17) $$\begin{array}{l} RMSE=\sqrt{\frac{1}{n}\overset{n}{\underset{i=1}{\sum }}{\left(y_{L}^{i}-y_{B_{*}}^{i}\right)}^{2}} \end{array}$$

where, *i*, index of data point; *y*_*L*_, original transformed Loihi values; *y*_B_*__, parameter-altered BMTK values; *n*, number of data points.

We repeat the same experiment in the other direction. We keep the parameters in the BMTK model the same, however we alter the parameters in the Loihi model one at a time. The current RMSE is given as:


(18) $$\begin{array}{l} RMSE=\sqrt{\frac{1}{n}\overset{n}{\underset{i=1}{\sum }}{\left(y_{L_{*}}^{i}-y_{B}^{i}\right)}^{2}} \end{array}$$

where, *i*, index of data point; *y*_L_*__, parameter-altered Loihi values; *y*_*B*_, original BMTK values; *n*, number of data points.

#### 3.1.2. Two-at-a-time method

We extend our sensitivity analysis a step further by assessing the effect of the interaction of two variables on the model output. Hence, to analyze the simultaneous impact of more than one variable, we implement a variation of the OAT method. In other words, we alter two parameters simultaneously rather than one at a time. We call this the two-at-a-time (TAT) method. This method helps us assess how the parameters affect the model when paired together as well as determine which variable is more susceptible to inducing change.

Let **A** = {*a*_1_, *a*_2_, ..., *a*_*n*_} and **B** = {*b*_1_, *b*_2_, ..., *b*_*m*_} be the two sets that represent the permitted values for the two parameters. Thus, implementing TAT involves drawing out an ordered pair (*a*_*p*_, *b*_*q*_) from the Cartesian product of {**A** × **B**}.

Thus, for every (*a*_*p*_, *b*_*q*_) ∈ {**A** × **B**}, we run the simulation. As in the OAT, we run the simulation in both directions. That is—in the first direction we keep the Loihi values fixed and transform the values for BMTK. In the second direction, we keep the BMTK values fixed but alter only the Loihi values.

Thus in the first run, the new RMSE looks as follows:


(19) $$\begin{array}{l} RMSE=\sqrt{\frac{1}{n}\overset{n}{\underset{i=1}{\sum }}{\left(y_{L}^{i}-y_{B_{**}}^{i}\right)}^{2}} \end{array}$$

where, *i*, index of data point; *y*_*L*_, original transformed Loihi values; *y*_B_**__, parameter-altered BMTK values; *n*, number of data points.

We repeat the simulation in the other direction. We keep the parameters in the BMTK model fixed as the original, however we alter the parameters in the Loihi model two at a time. The current RMSE is given as:


(20) $$\begin{array}{l} RMSE=\sqrt{\frac{1}{n}\overset{n}{\underset{i=1}{\sum }}{\left(y_{L_{**}}^{i}-y_{B}^{i}\right)}^{2}} \end{array}$$

where, *i*, index of data point; *y*_L_**__, parameter-altered Loihi values; *y*_*B*_, original BMTK values; *n*, number of data points.

In the next section, we illustrate the results obtained by implementing the models on the two platforms and their subsequent validation based on the methods mentioned in this chapter.

As done for the mapping validation in the previous chapter, we run the analysis for both network inputs: External Spikes and Bias Current. It is worth reiterating here that when we analyse the BMTK results, we do it against the validated fixed Loihi model and while performing the analysis on Loihi, we check the results against the validated fixed BMTK models. We do so as this helps us verify our results against previously validated models which form the foundation for these findings.

### 3.2. Perturbation and sensitivity analysis on BMTK

We explore the sensitivity of stimulation outcomes generated by two different method of stimulating the studied neural models: external spikes, and bias currents. We keep the External Spikes stimulus (Table 1) and the bias current stimulus (200.0 *pA*) same as used during the validation of the networks. It is worth noting here that the RMSE for these networks are calculated only over the subthreshold trajectories of the voltage trace to ensure the spike timing does not contribute to the discrepancies in the microstate cost function. The spike-based cost function currently is beyond the scope of this work.

#### 3.2.1. One-at-a-time method

We start the analysis with the one-at-a-time (OAT) method for BMTK. Here, we perturb one parameter at a time while keeping the other parameters same as their original value.

##### 3.2.1.1. Effect of membrane capacitance (*C*_*m*_)

**Figure 1:**
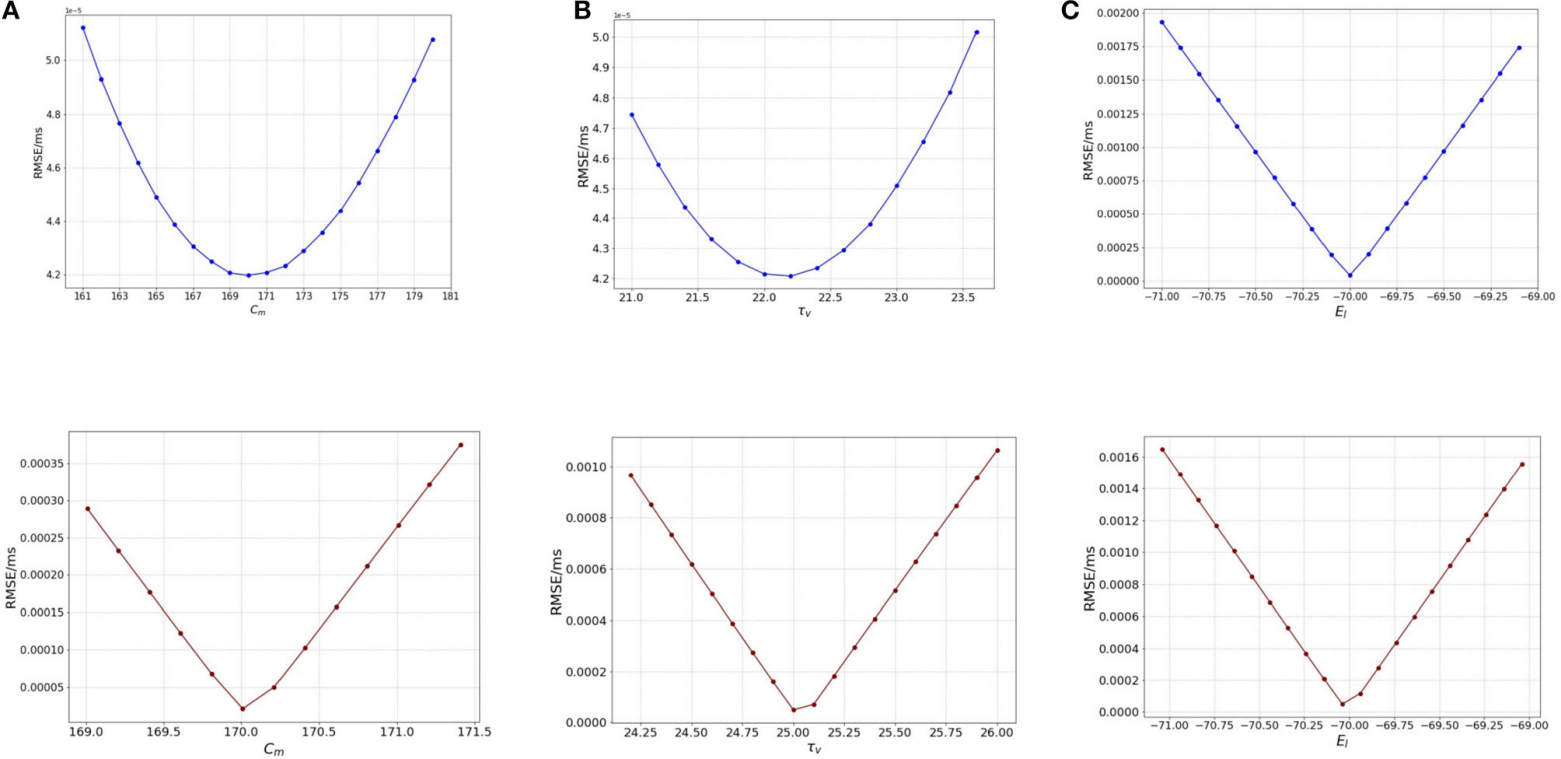
Plot showing the RMSE values based on OAT perturbations of different parameters. Blue curves show results for external spike inputs; red curves show results for simulations with bias current. **(A)** Various membrane capacitance *C*_*m*_ values; **(B)** various membrane time constant τ_*v*_ values; and **(C)** various resting potential *E*_*L*_ values.

We start the analysis with membrane capacitance. As seen in Table 1, the original model in the study has a value 170.0 pF. To analyse the sensitivity of the model, we work with a range of values for *C*_*m*_. We run the model for each of the values in the membrane capacitance range while keeping the other parameter and state values the same. The range of the parameter is set as [160, 180] (pF) with a step-size of 1.0. We choose this range as this allows for good scope for perturbation while being computationally economical. Figure 1A (blue) illustrates the RMSE values versus the range of values in *C*_*m*_ for external spikes.

We once again perturb the parameters *C*_*m*_ for this network with bias input. The range for perturbation used here is smaller to highlight the effect of bias current as compared to the external spikes. As we vary the membrane capacitance for the bias-current run network, we observe very distinct characteristics of the Figure 1A (red), and find that it is slightly different from the spike-based network. The range considered here is [169, 171.5] with a step-size of 0.2. We find that the minimum for RMSE (4.208 × 10^−5^ mV/ms) exists at the original value of 170.0 pF (Table 1). It can be inferred that *C*_*m*_ affects the model to some extent, i.e., with proposed change in the values of *C*_*m*_, the RMSE changes a fair amount.

The lowest RMSE is the same for both stimuli as seen during the validation with a value of 1.1474 × 10^−4^ mV/ms found at 170.21 pF, while the maximum value goes to 3.5 × 10^−4^ mV/ms found around 171.25 pF. Local range perturbations also help in keeping the simulations computationally inexpensive.

##### 3.2.1.2. Effect of membrane time constant (τ_*v*_)

Next, we perform the same analysis based on membrane time constant (τ_*v*_), i.e., we alter the values of τ_*v*_ for each run of the model while keeping the other parameters the same. The original value for τ_*v*_ based on Table 1 is 22.2 ms. We set the range for the analysis as [21.0, 23.6] (*ms*) for external spikes and [24.0, 26.2] for bias current with a step-size of 0.2 for both.

Figure 1B illustrates the RMSE values versus the range of τ_*v*_ values. It can be seen that minimum is at 22.2 ms, the same as found for the validated models. However, the rise in the RMSE values on either side of the minimum is relatively gradual, and the maximal values on the boundary are comparable to those of *C*_*m*_. Thus, it can be inferred here that the impact of τ_*v*_ and *C*_*m*_ on the model are relatively close.

Similar to membrane capacitance *C*_*m*_, we find that τ_*v*_ for bias current behaves somewhat differently from its spike-run counterpart. Figure 1B illustrates the variation of the RMSE as vary the parameter. The sharp local minimum is found as for the validated networks. On either side of the minimum, the curves rises steeply, similar to the behavior seen in *C*_*m*_, however the change in RMSE is relatively lesser (1.0 × 10^−4^ for τ_*v*_ and 3.5 × 10^−4^ for *C*_*m*_).

##### 3.2.1.3. Effect of resting potential (*E*_*L*_)

The default value for the resting potential (*E*_*L*_) in the model is set at −70.0 mV. For the sensitivity analysis, the value for *E*_*L*_ is varied in the range of [−71.0, −69.0] mV with a step-size of 0.1. This range allows a good visual of the effect of (*E*_*L*_) on the RMSE of the model. The effect is seen to be very stark, i.e., even for a slight variation from −70.0 mV which is the original value for the model, we see a huge change in the RMSE value (which is 0.002 mV/ms) as compared to for *C*_*m*_ and τ_*v*_ (at 0.8 × 10^−5^ mV/ms).

Figure 1C illustrates the variation of the RMSE values for a range of the *E*_*L*_ values. The minimum is found at −70.0 mV, the original value for the model and we see that a slight variation on either side of the default value causes a steep difference in the RMSE, the maximum RMSE being as high as 0.002 mV/ms. This observation indicates that *E*_*L*_ is a highly sensitive parameter and affects the model greatly. A larger range of values yields the same results.

For bias input, we vary the values for *E*_*L*_ in the same range as we do for the spike-run network. Figure 1C illustrates the variation of RMSE as we vary *E*_*L*_. We use the range for *E*_*L*_ as [−71.0, −69.0.] with a step-size of 0.1. We find that the behavior of the curve looks similar to the spike-run network, with the minimum found with a steep drop in the curve at −70.04 mV. The change in RMSE here is significant (at 0.0016 mV/ms) as compared to the ones seen for *C*_*m*_ and τ_*v*_ implying *E*_*L*_ is the most impactful parameter for the model.

#### 3.2.2. Two-at-a-time method, spike input

Next, we perform the two-at-a-time (TAT) analysis. Here, we perturb two parameters together, and keep the rest of the parameter values unchanged. We work with the following combinations: (1) {*C*_*m*_, τ_*v*_ }, (2) {*C*_*m*_, *E*_*L*_}, and (3) {*E*_*L*_, τ_*v*_}.

##### 3.2.2.1. Effect of membrane capacitance and membrane time constant {*C*_*m*_, τ_*v*_}

In the model, the original values for membrane capacitance (*C*_*m*_) and membrane time constant (τ_*v*_) are 170.0 pF and 22.2 ms, respectively. For the analysis, we work on a range of [160, 180] for *C*_*m*_ with a step-size of 1.0 and [21.0, 23.6] for τ_*v*_ with a step-size of 0.2, almost the same range as done for the OAT method.

Based on the method described above, we consider a point from the range of *C*_*m*_ and τ_*v*_ each, and implement a run for each of the combination of values i.e., (*a*_*i*_, *b*_*j*_) ∈ {**A** × **B**}. Here, **A** is the set of *C*_*m*_ values and **B** is the set of τ_*v*_ values.

**Figure 2:**
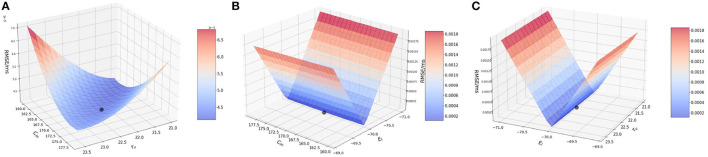
Plot showing the RMSE values based on the combination of. **(A)** *C*_*m*_ and τ_*v*_ values; **(B)** *C*_*m*_ and *E*_*L*_ values; and **(C)** τ_*v*_ and *E*_*L*_ values.

Figure 2A illustrates the variation of RMSE as we vary the *C*_*m*_ and τ_*v*_ values simultaneously. We find surface in which the curvature is slightly more significant on the *C*_*m*_ axis with a high RMSE found near the extremities of *C*_*m*_ values, with a dip near {*C*_*m*_, τ_*v*_} values of 170.0 pF and 22.2 ms respectively.

It can be inferred that even during the interaction between the two parameters, the results from the OAT method hold.

##### 3.2.2.2. Effect of membrane capacitance and resting potential {*C*_*m*_, *E*_*L*_}

For this section, we work with membrane capacitance *C*_*m*_ and resting potential *E*_*L*_. The original values for these two parameters is 170.0 pF and −70.0 mV, respectively. We vary *C*_*m*_ as before in the range of [160, 180] with a step-size of 1.0 and for *E*_*L*_, we vary it in the range of [−71.0, −69.0] with a step-size of 0.1. The choice for the ranges is kept similar to the OAT method, and we find that variation over this range provides us with good evidence of the sensitivity of the parameters.

In Figure 2B, we observe that the slope is very steep on the *E*_*L*_ axis, whereas for *C*_*m*_ the gradient is barely visible and it is seen close to 170.0 pF. We can attribute this characteristic of the curve to the impact that *E*_*L*_ has on the RMSE as compared to *C*_*m*_. As found numerically, the highest and lowest RMSE for *E*_*L*_ are 0.002 mV/ms and 4.208 × 10^−5^ mV/ms respectively, whereas for *C*_*m*_, the highest and lowest values are much smaller i.e., 5.0 × 10^−5^ mV/ms and 4.208 × 10^−5^ mV/ms respectively.

Thus, the LIF models is far more sensitive to changes in *E*_*L*_ than to changes in *C*_*m*_ and for the interaction of these two parameters, the lowest RMSE is achieved for the values of *E*_*L*_ as −70.0 mV and for *C*_*m*_ as 170.0 pF. Accordingly, an initial optimization along the *E*_*L*_ variable is more likely to produce high quality results for fixed *C*_*m*_.

##### 3.2.2.3. Effect of membrane time constant and resting potential {τ_*v*_, *E*_*L*_}

In this section, we examine the interaction of membrane time constant (τ_*v*_) and resting potential (*E*_*L*_) values. As mentioned earlier, the original values used in the model are 22.2 ms and 170.0 pF, respectively. We vary the ranges similar to the previous two simulations.

Figure 2C illustrates the interaction between τ_*v*_ and *E*_*L*_. We observe that the plot is very similar to Figure 2B. The reason being, *E*_*L*_ once again impacts the model far more than τ_*v*_. Numerically, the highest and lowest RMSE for *E*_*L*_ are 0.002 and 4.208 × 10^−5^ mV/ms respectively, whereas for τ_*v*_, the highest and lowest values are much smaller i.e., 5.0 × 10^−5^ and 4.208 × 10^−5^ respectively.

Thus, from the above simulation it can be concluded that *E*_*L*_ affects the model to the greatest extent whereas *C*_*m*_ and τ_*v*_ have a much smaller effect on the model. Thus, effect on the parameters on the model can ranked as *E*_*L*_ >> *C*_*m*_ ≈ τ_*v*_.

#### 3.2.3. Two-at-a-time method, bias input

In this section, we perform the two-at-a-time (TAT) analysis. As done earlier, we perturb two parameters together, and keep the rest of the parameter values unchanged. We work with the following combinations : (1) {*C*_*m*_, τ_*v*_ }, (2) {*C*_*m*_, *E*_*L*_}, and (3) {*E*_*L*_, τ_*v*_}.

##### 3.2.3.1. Effect of membrane capacitance and membrane time constant {*C*_*m*_, τ_*v*_}

We vary the membrane capacitance *C*_*m*_ over the range of [169, 171.5] with a step-size of 0.2 and membrane time constant τ_*v*_ in the range of [24.0, 26.2], again with a step-size of 0.2 The plot nature closely follows the features displayed by the *C*_*m*_ and τ_*v*_ OAT figures.

**Figure 3:**
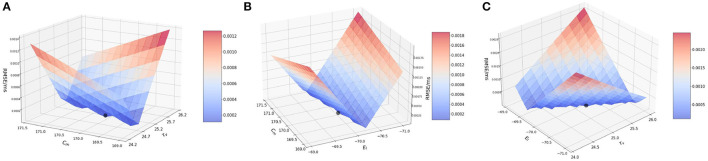
Plot showing the RMSE values based on the combination of **(A)** *C*_*m*_ and τ_*v*_ values; **(B)** *C*_*m*_ and *E*_*L*_ values; and **(C)** τ_*v*_ and *E*_*L*_ values.

Figure 3A illustrates the surface obtained by varying the two parameters simultaneously. Similar to the OAT plots for *C*_*m*_ and τ_*v*_, the new graph encompasses the behavior of the two parameters, with the RMSE minimum found at the same locations as 170.21 pF and 25.1 ms. As the error values are very small, graph interpretation is slightly challenging and thus, we verify the value of the minimum numerically which matches the original minimum of 1.1474 × 10^−4^ mV/ms found for the validation networks.

##### 3.2.3.2. Effect of membrane capacitance and resting potential {*C*_*m*_, *E*_*L*_}

Next, we run the analysis for a bias-induced network while varying membrane capacitance *C*_*m*_ and resting potential *E*_*L*_ together.

Figure 3B illustrates the variation of RMSE as we perturb the parameters. It can be observed that this plot captures the curves and the lowest point as found in the *C*_*m*_ and *E*_*L*_ plots. The minimum is found at 170.21 pF and around −70.04 mV.

##### 3.2.3.3. Effect of membrane time constant and resting potential {τ_*v*_, *E*_*L*_}

As seen in the earlier figures, the TAT plots follow the OAT curves closely. We can see a similar attribute while varying membrane time constant and resting potential simultaneously.

Figure 3C illustrates the RMSE values while perturbing these two parameters. As can be seen from the figure, the lowest RMSE is found close to the original value at 25.1 ms and −70.0 mV.

#### 3.2.4. Conclusion: BMTK simulations validate Proposition 1 and Proposition 2

- **Proposition 1:** *Based on our analysis of our* ℓ_2_*-cost function, we had proposed that the RMSE values obtained during the validation process serve as the global minimum. Additionally, larger perturbation leads to a significant deviation from this minimum*.
  From the simulation results we find that the RMSE values obtained during validation serve as our global minimum, with no other minima found for the different values.
  Also, we see a sharp increase in the *y*-axis values as the perturbation steps increase. For *E*_*L*_, we see an almost exponential rate of change. For *C*_*m*_ and τ_*v*_ though the rate is slower, a larger increase is still seen as we move away from the minimum found.
- **Proposition 2:** *We used derivative-analysis to establish that perturbing E*_*L*_ *impacts the cost function most significantly*.
  It can be observed from the sensitivity analysis results for BMTK that *E*_*L*_ affects the cost function RMSE the most, followed by τ_*v*_ and *C*_*m*_, which is the result we found using the derivative-analysis. Thus, *E*_*L*_ has the maximum impact on the RMSE with the lowest perturbation step-size leading to almost 42% increase in the error while for τ_*v*_ and *C*_*m*_ it is as low as 0.2%. Thus, τ_*v*_ and *C*_*m*_ are seen to have a smaller impact for both spike induced and bias-current induced networks as compared to the more sensitive *E*_*L*_, with the results holding for simultaneous variations where τ_*v*_ and *C*_*m*_ interact with *E*_*L*_.

### 3.3. Perturbation and sensitivity analysis on Loihi

As done in BMTK, we start the analysis with the one-at-a-time (OAT) method for Loihi. It is to be noted here that the state parameters and state variables in Loihi work differently as compared to BMTK, i.e., the state parameters *C*_*m*_, τ_*v*_, and *E*_*L*_ do not contribute to the model directly but as a combination of several other values which ultimately form the different state variables for the network. Moreover, those values are stored as integers, which can restrict their precision. It is worth mentioning here that the plots below are slightly “unsmooth” as compared to BMTK. This effect can be attributed to discretization error when those values are stored in Loihi with integer rounding, thus storing the values slightly differently.

In Loihi, we use the same External Spikes stimulus (Table 1) used during the validation of the networks, translated to Loihi timebase. We also repeat the same analysis as above with the network stimulated with bias current, i.e., *I*_*e*_ ≠ 0. Based on our BMTK values, we use *I*_*e*_ = 200.0*pA*, same as used during the validation of the networks. We once again perturb our same set of parameters.

#### 3.3.1. One-at-a-time method

We start with the OAT method as done for BMTK, by varying one parameter at a time and keep the rest of the parameters the same. As discussed earlier in Chapter 3, the equations given below are the governing equations for our analysis on Loihi:


(21) $$\begin{array}{l} v\left(t+1\right)=v\left(t\right)\left[1-\frac{\delta_{v}}{2^{12}}\right]+b+u\left(t\right), \end{array}$$

(22) $$\begin{array}{l} u\left(t\right)=\frac{1}{CV_{s}}I_{e}\left(t\right)+\frac{1}{\tau_{v}}\frac{E_{L}-V_{r}}{V_{s}} \end{array}$$

where *C* denotes the membrane capacitance *C*_*m*_ as seen in BMTK, *b* is the constant bias current and δ_*v*_ is the voltage decay parameter used as $\delta_{v}=\frac{dt}{\tau_{v}}\;2^{12}$ in the model.

##### 3.3.1.1. Effect of membrane capacitance (*C*_*m*_)

We begin with examining the effect of *C*_*m*_ on the model. As seen from Eqs. (21) and (22), *C*_*m*_ as a parameter contributes to the model dynamics directly in terms of the state variable *u*(*t*), the synaptic current/bias current depending on how the network is established, and indirectly through *v*(*t*) as $\delta_{v}=\frac{dt}{\tau_{v}}\;2^{12}=\frac{dt}{RC}\;2^{12}$.

As we run the network with external spikes, *I*_*e*_(*t*) = 0.0 based on the LIF parameters, thus *u*(*t*) = 0. But we observe the effect of *C*_*m*_ on the RMSE with the help of Equation (21). We set *R* as a constant with the help *R* = τ_*v*_/*C*_*m*_ = 22.2/170.0, which are the original values used for τ_*v*_ and *C*_*m*_ in the model.

**Figure 4:**
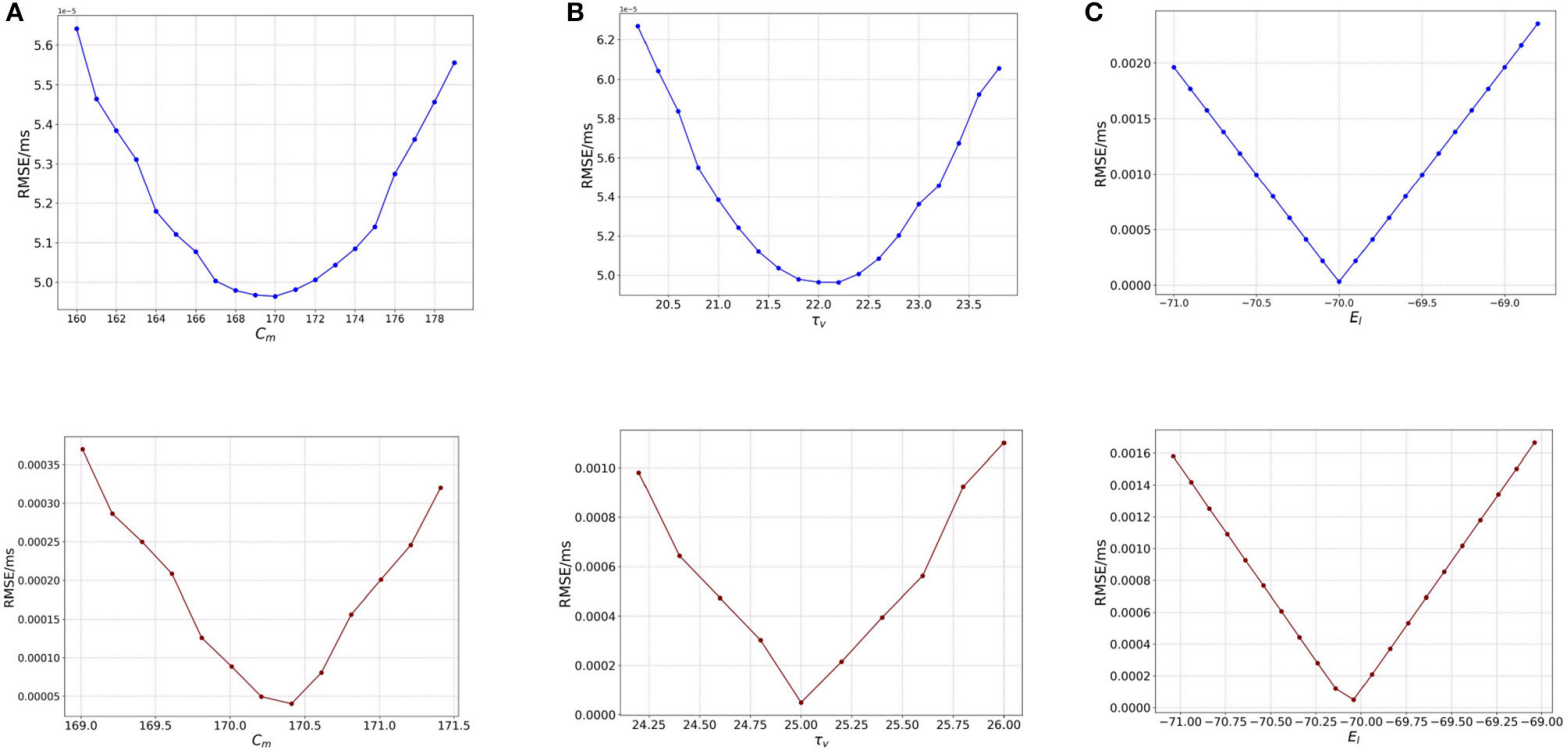
Plot showing the RMSE values based on OAT perturbations of Loihi parameters. Blue curves show results for external spike inputs; red curves show results for simulations with bias current. **(A)** Various membrane capacitance *C*_*m*_ values; **(B)** various membrane time constant τ_*v*_ values; and **(C)** various resting potential *E*_*L*_ values.

For the analysis we vary the value of *C*_*m*_ over the same range as we did for BMTK—[160, 180] (pF) with a step-size of 1.0. As seen in Figure 4A (blue), we notice that the RMSE behaves very similar to the variation of *C*_*m*_ in BMTK [Figure 1A (blue)], with a minimum obtained at 170.0 pF.

We asses the impact of the parameter for a bias-current induced network, i.e., where *I*_*e*_ ≠ 0. We start with the OAT method as done for BMTK, by varying one parameter at a time. Figure 4A (red) illustrates the variation of RMSE as we perturb *C*_*m*_. The minimum here is found to be the same as used for the validation network at 170.21 pF. Also, it can be seen that the minimum is clearly designated with the RMSE on either side of it increasing to a fair extent. Thus, *C*_*m*_ has a reasonable impact on the RMSE.

##### 3.3.1.2. Effect of membrane time constant (τ_*v*_)

To analyse the effect of τ_*v*_ on the model, we refer back to Equation (21) and the definition of δ_*v*_ which denotes that τ_*v*_ contributes to the Loihi voltage decay parameters inversely by a scale of *dt* * 2^12^. As seen in the case of *C*_*m*_, regardless of *u*(*t*) being absent here, we see a variation for τ_*v*_ as it is directly related to the evolution of the membrane potential *v*(*t*).

The original values for τ_*v*_ is 22.2 ms and for the analysis we vary it over a range of - [19.0, 23.6] (*ms*) with a step-size of 0.2.

Figure 4B (blue) illustrates the RMSE values as we vary the τ_*v*_ values for external spikes. The minimum is achieved for τ_*v*_ = 22.0 which is exactly the same as the BMTK minimum [Figure 1B (blue)]. Thus, τ_*v*_ behaves similarly for BMTK and Loihi.

For bias inputs, we vary the membrane time constant τ_*v*_ over the range of [23.2, 26.0] (*ms*) with a step-size of 0.2. Once again, we focus on a shorter range to highlight the effect of τ_*v*_ on RMSE in spite of a small variation. Figure 4B (red) illustrates the variation of RMSE for different values of τ_*v*_ for bias current. The minimum is found at 22.2, which is same as the value found for the validation networks. Also, the minimum found for τ_*v*_ is quite sharp with steep increase in the RMSE on either side of it, however compared to *C*_*m*_, the change in the RMSE values is smaller. This implies τ_*v*_ has an effect on the RMSE which is slightly more than *C*_*m*_.

##### 3.3.1.3. Effect of resting potential (*E*_*L*_)

Similar to *C*_*m*_, *E*_*L*_ also features in the equation of *u*(*t*). As *I*_*e*_ = 0.0, the new equation for *u*(*t*) looks as follows :


(23) $$\begin{array}{l} u\left(t\right)=\frac{1}{\tau_{v}}\frac{E_{L}-V_{r}}{V_{s}} \end{array}$$

and since we vary *E*_*L*_, *E*_*L*_ − *V*_*r*_ ≠ 0 except at one point. Thus, *u*(*t*) still features in the model equation and contributes to the dynamics, unlike the case for *C*_*m*_.

Here, we consider the same range of [−71.0, −69.0] with a step-size of 0.1 for *E*_*L*_, as done for BMTK. Figure 4C illustrates the variation of RMSE as we vary *E*_*L*_. We find the behavior to be the same as seen in BMTK with a sharp minimum observed at the point −70.0 mV, which is the value obtained for the validated networks. As can be seen from the figures above, similar to BMTK, *E*_*L*_ affects the cost function the most, with relatively smaller contribution by *C*_*m*_ and τ_*v*_.

We repeat our analysis for resting potential *E*_*L*_ for bias inputs, over the range of [−71.0, −69.0] with a step-size of 1.0. We find that the behavior resembles that of the BMTK analysis. Figure 4C (red) represents the different values of RMSE as we change the values of *E*_*L*_ for bias current. The lowest RMSE is found at the original value of −70.04 mV. However, it is worth noting here that for a slight variation in *E*_*L*_, the RMSE increases significantly which is much higher as compared to *C*_*m*_ and τ_*v*_.

#### 3.3.2. Two-at-a-time method, spike input

Next, we perform the two-at-a-time (TAT) analysis. Here, we perturb two parameters together, and keep the rest of the parameter values unchanged. We work with the combinations as previously done for BMTK : (1) {*C*_*m*_, τ_*v*_ }, (2) {*C*_*m*_, *E*_*L*_}, and (3) {*E*_*L*_, τ_*v*_}.

##### 3.3.2.1. Effect of membrane capacitance and membrane time constant {*C*_*m*_, τ_*v*_}

We vary *C*_*m*_ and τ_*v*_ on the ranges [160, 180] (*pF*) with a step-size of 1.0 and [19.0, 23.6] (*ms*) with a step-size of 0.2 respectively. As seen in BMTK, the TAT simulations closely follow the simulations based on OAT.

It is worth noting here that for the analysis of *C*_*m*_ in the OAT method, we fixed our *R* to a value obtained by the original values of *C*_*m*_ andτ_*v*_, and for the OAT analysis of τ_*v*_, the variation of τ_*v*_ was done directly with the help of δ_*v*_, the voltage decay parameter. In the TAT case however, we work a little differently as τ_*v*_ and *C*_*m*_ are updated simultaneously, thus impacting the values of *R*, τ_*v*_, and *C*_*m*_ for each iteration.

**Figure 5:**
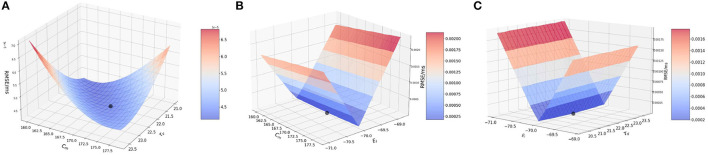
Plot showing the RMSE values during bias current simulations based on the combination of **(A)** *C*_*m*_ and τ_*v*_ values; **(B)** *C*_*m*_ and *E*_*L*_ values; and **(C)** τ_*v*_ and *E*_*L*_ values.

Figure 5A illustrates the variation in the cost function as we vary the two parameters. As can be seen from the figure, effects of the changes in the values of *C*_*m*_ and τ_*v*_ produces similar effects on the RMSE. This implies that *C*_*m*_ and τ_*v*_ contribute closely to changes in RMSE. The lowest RMSE is found for *C*_*m*_ = 170.0 and τ_*v*_ = 22.0, which again reiterates the OAT result.

##### 3.3.2.2. Effect of membrane capacitance and resting potential {*C*_*m*_, *E*_*L*_}

We vary *C*_*m*_ and *E*_*L*_ over the same ranges as before—[160, 180](*pF*) with a step-size of 1.0 and [−71.0, −69.0] with a step-size of 0.2, respectively. As seen for the OAT results, we expect the RMSE to vary more significant on the *E*_*L*_-axis than the *C*_*m*_-axis.

Figure 5B illustrates the RMSE values for this combined perturbation. Very little variation if found with respect to *C*_*m*_ as the error values contributed by *C*_*m*_ (change of 1.5 × 10^−5^ mV/ms) are relatively smaller compared to *E*_*L*_ (change of ~0.0025 mV/ms). The minimum is found at *C*_*m*_=170.0 pF. and *E*_*L*_ = −70.0 mV.

##### 3.3.2.3. Effect of membrane time constant and resting potential {τ_*v*_, *E*_*L*_}

We vary τ_*v*_ and *E*_*L*_ over the ranges of [19.0, 23.6] (*ms*) with a step-size of 0.2 and [−71.0, −69.0] with a step-size of 0.1 respectively, as done earlier.

Figure 5C illustrates the variation of RMSE. As seen in Figure 5A—for the combination of *C*_*m*_ and τ_*v*_, the variation is seen more prominently only on the *E*_*L*_ axis as compared to τ_*v*_ remains constant. The minimum is found for *E*_*L*_ = −70.0 mV and τ_*v*_ = 22.0 ms.

This completes our evaluation of sensitivity of the model to the state parameters based on spike-run networks. Thus, effect on the parameters on the model can ranked similar to our observation in BMTK, i.e., *E*_*L*_ >> *C*_*m*_ ≈ τ_*v*_.

#### 3.3.3. Two-at-a-time method, bias input

Finally, we perform the two-at-a-time (TAT) analysis which forms the last section of our sensitivity analysis of these three state parameters. Here, we perturb two parameters together, and keep the rest of the parameter values unchanged. We work with the combinations as previously done for BMTK : (1) {*C*_*m*_, τ_*v*_ }, (2) {*C*_*m*_, *E*_*L*_}, and (3) {*E*_*L*_, τ_*v*_}.

##### 3.3.3.1. Effect of membrane capacitance and membrane time constant {*C*_*m*_, τ_*v*_}

We vary *C*_*m*_ and τ_*v*_ over the same range as done for OAT. Since the TAT simulations are closely governed by the OAT ones, we find that in this case too the simulations follow the OAT results.

**Figure 6:**
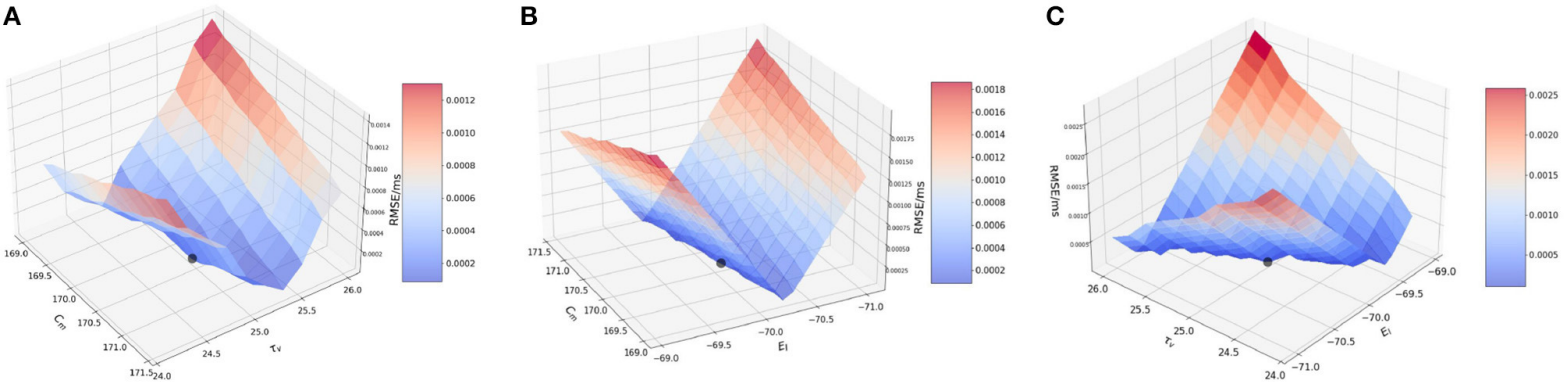
Plot showing the RMSE values during bias current simulations based on the combination of **(A)** *C*_*m*_ and τ_*v*_ values; **(B)** *C*_*m*_ and *E*_*L*_ values; and **(C)** τ_*v*_ and *E*_*L*_ values.

Figure 6A illustrates the result of varying *C*_*m*_ and τ_*v*_ simultaneously. It closely follows the BMTK result with a minimum found at 170.21 pF for *C*_*m*_ and 25.1 ms for τ_*v*_. As the plot representation doesn't pinpoint the exact position, the values have been numerically verified.

##### 3.3.3.2. Effect of membrane capacitance and resting potential {*C*_*m*_, *E*_*L*_}

Next, we vary *C*_*m*_ and *E*_*L*_ over the range of [169.0, 171.0] (pF) with a step-size of 0.2 and [−71.0, −69.0](*mV*) with a step-size of 0.1.

Figure 6B illustrates the variation of RMSE based on varying *E*_*L*_ and *C*_*m*_. The plot closely resembles that of BMTK, the minimum is reached at the same points : −70.04 mV for *E*_*L*_ and close to 170.21 pF for *C*_*m*_. The results closely follow the minimum found for the OAT results.

##### 3.3.3.3. Effect of membrane time constant and resting potential {τ_*v*_, *E*_*L*_}

Finally, we vary τ_*v*_ and *E*_*L*_ simultaneously over the range of [24.0, 26.2] (*ms*) with a step-size of 0.2 and [−71.0, −69.0] with a step-size of 0.1, respectively.

Figure 6C illustrates the effect of varying τ_*v*_ and *E*_*L*_ simultaneously. As seen from the plot and from numerical verification, the lowest RMSE is obtained for τ_*v*_ close to 25.1 ms and *E*_*L*_ close to −70.04 mV, which is comparable to what we observe for the validation networks.

Thus, based on the last six findings, we deduce that *E*_*L*_ contributes most significantly to the RMSE output with sharp increase in its values with a slight variation of *E*_*L*_. It is closely followed by *C*_*m*_ and then by τ_*v*_ which contributes to the least amount of changes in the RMSE values.

This brings us to the end of analyzing the sensitivity of the cost function to the variations of the state parameters for both BMTK and Loihi. We find that BMTK and Loihi results closely match each other, with lowest RMSE found at similar positions for both. We reiterate here that we focus on focused local ranges for the parameter variations to assess the change in cost function with small perturbation and also to avoid high computational expenses. Moreover, global sensitivity analysis would require further investigation which at present is beyond the scope of this work.

#### 3.3.4. Conclusion: Loihi simulations validate Proposition 1 and Proposition 2

As done for BMTK, we verify Propositions 1 and 2 for Loihi.

- **Proposition 1:** *RMSE values obtained during the validation process serve as the global minimum. Secondly, larger perturbation leads to a significant deviation from this minimum*.
  Since Loihi follows the BMTK results closely, we find that statements in Proposition 1 are satisfied in a similar fashion as in BMTK, i.e., from the simulation results we find that the RMSE values obtained during validation serve as our global minimum. Moreover, these values are the same as seen for BMTK.
- **Proposition 2:** *Using derivative-analysis, we find that perturbing E*_*L*_ *impacts the cost function most significantly*.
  As dictated by the results in BMTK, for Loihi too we find the most impact contributed by *E*_*L*_, for both external spikes and bias current stimuli. Although *C*_*m*_ and τ_*v*_ seem to affect the results relatively more for bias current induced stimuli, they do not supersede the impact of *E*_*L*_.
  For external-spikes induced network, *E*_*L*_ has the maximum impact on the RMSE with the lowest perturbation step-size leading to almost 42% increase in the error while for τ_*v*_ and *C*_*m*_ it is as low as 0.2%. However, for bias-current based network, second perturbation leads to similar increase in the RMSE values for both *E*_*L*_ and τ_*v*_ (impact of *C*_*m*_ is much lower), however, as the perturbation steps increase the maximum impact is seen to be contributed by *E*_*L*_. Thus, τ_*v*_ and *C*_*m*_ are seen to have a smaller impact for both spike induced and bias-current induced networks as compared to the more sensitive *E*_*L*_, with the results holding for simultaneous variations where τ_*v*_ and *C*_*m*_ interact with *E*_*L*_.

### 3.4. Perturbation and sensitivity analysis on Loihi based on parameter precision levels

In the following sections, we investigate two values that are combinations of the parameters we dealt with above. As these variables allow us control over the different parameters, our goal is to examine how changes in their precision affect the cost function RMSE.

#### 3.4.1. Effect of bias current (*I*_*bias*_)

Since *C*_*m*_ and *E*_*L*_ contribute to the formation of the state variable *u*(*t*) in Loihi which is the state current/bias current represented as *I*_*bias*_, we wanted to check how varying *I*_*bias*_ directly affects the RMSE values for the model. Does it show equivalence to the results found in by the variations in *C*_*m*_ and *E*_*L*_?

*I*_*bias*_ is expressed as a combination *I*_*bias*_ mantissa and *I*_*bias*_ exponent values. This allows for a large range of values to be included in the simulations. The bias mantissa is allowed a range between [−2^12^, 2^12^] and the bias exponent a range between [0, 7]. Thus, for our simulation we vary the bias exponent over the entire allowed range of [0,7] while simultaneously adjusting (dividing) the bias mantissa by a factor of 2^*i*^, where *i* ∈ [0, 7].

The Loihi value of *I*_*bias*_ obtained by applying the transformation (Equation 6) on the BMTK *I*_*e*_(*t*) value of 200.0*pA* is 1175.0. We take it as the base value and then vary the precision allowed by the bias exponent in Table 2 to investigate how it contributes to the variation in the value of *I*_*bias*_ and thus, ultimately to RMSE. It is worth noting that *I*_*bias*_ can only be use as an integer-value in Loihi.

**Table 2:** Effect of precision on *I*_*bias*_.

** $a=I_{bias}/2^{i}$ **	**Bias exponent (*i*)**	Reconstructed $I_{bias}=a*2^{i}$
1,175	0	1,175
587	1	1,174
293	2	1,172
146	3	1,168
73	4	1,168
36	5	1,152
18	6	1,152
9	7	1,152

Values in the first and third column are calculated as integers, the data-type used by Loihi for *I*_*bias*_.

**Figure 7:**
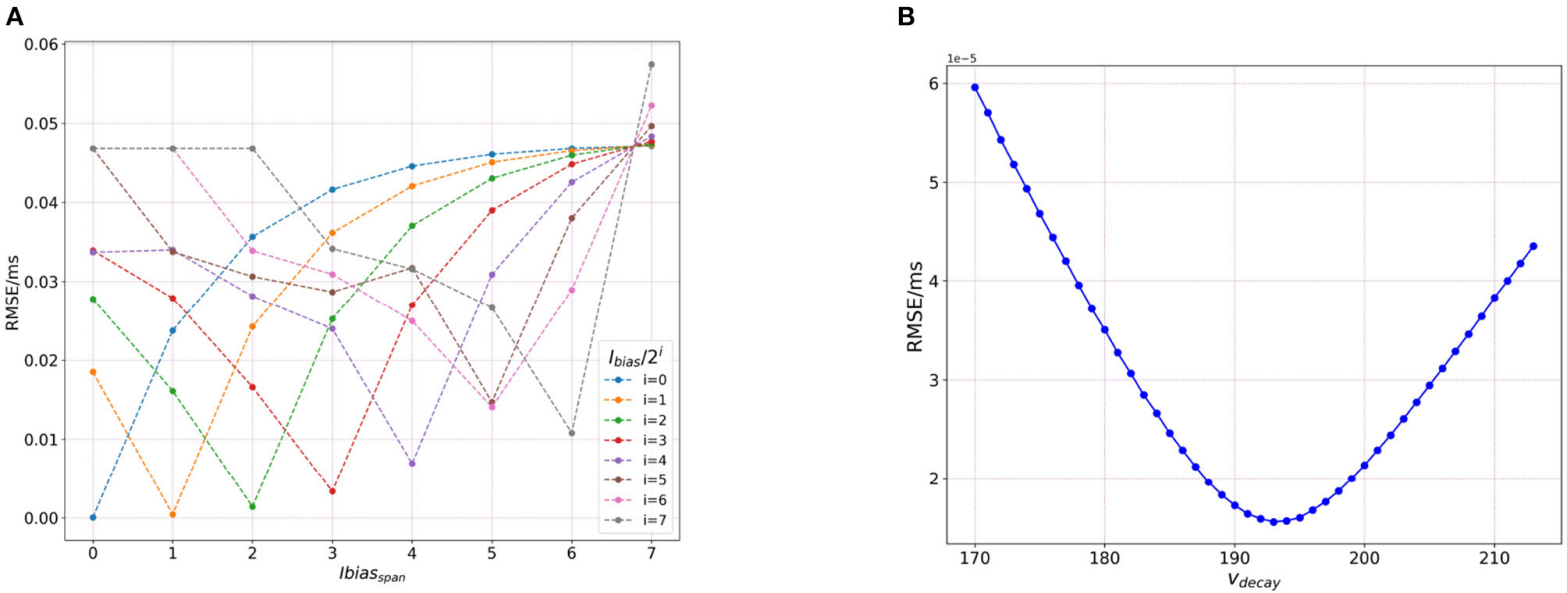
Plot showing sensitivity of Loihi simulations due to integer nature of parameters stored in Loihi. **(A)** The RMSE values based on the variation of bias exponent and **(B)** RMSE values based on the variation of δ_*v*_.

Figure 7A shows the effect of varying the *I*_*bias*_ over both the exponent and mantissa components. For the analysis here, we calculate the RMSE variation only for the subthreshold variation of the trajectory (bypassing the spiking effect of the bias current), as done for the sensitivity analysis on networks run by bias current. It can be seen that the finer precisions yield low errors. This can be justified by Table 2 below as we can see that as we increase the value of *i*, the value of *I*_*bias*_ changes with significant loss in recovering the original value.

#### 3.4.2. Effect of voltage decay (δ_*v*_)

As seen in the previous section, we vary *I*_*bias*_ directly to analyze its impact on the RMSE as compared to varying *C*_*m*_ and *E*_*L*_. We do a similar investigation in terms of τ_*v*_ by altering the values for δ_*v*_ directly as


(24) $$\begin{array}{l} \delta_{v}=\frac{dt}{\tau_{v}}\;2^{12}. \end{array}$$

Thus, we vary δ_*v*_ over the allowed range of ±30. Figure 7B shows the RMSE curve achieved by this variation. In the figure, the values for *v*_*decay*_ are obtained by adding ±30 to 184 which is the base δ_*v*_ value, found after converting the original BMTK membrane time constant value of 22.2 ms.

As can be seen from Figure 7B, the lowest RMSE is obtained at 193.0, and after reversing the transformation in above Equation (24), it yields 21.2 which is relatively close to the original BMTK value of 22.2. This small discrepancy can be attributed to the value being processed as an integer in Loihi which leads to a certain loss of precision. We run this analysis only for *dt* = 1.

## 4. Conclusions and discussion

Inspired by the brain, neuromorphic computing holds great potential in tackling tasks with extremely low power and high efficiency. Many large-scale efforts including the TrueNorth, SpiNNaker, and BrainScaleS have been demonstrated as a tool for neural simulations, each replete with its own strengths and constraints. Fabricated with Intel's 14 nm technology, Loihi is a forward-looking and continuously evolving state-of-the-art architecture for modeling spiking neural networks in silicon.

As opposed to its predecessors, Loihi encompasses a wide range of novel features such as hierarchical connectivity, dendritic compartments, synaptic delays, and programming synaptic learning rule. These features, together with solid SDK support by Intel, and a growing research community, make Loihi an effective platform to explore a wealth of neuromorphic features in more detail than before.

In this study, we analyzed the sensitivity of the model cost function (based on the ℓ_2_-norm) to the different parameters of the neural model. In other words, we investigate how changing the state parameters of the LIF model affects the validation outcomes obtained previously and establish results that characterize the error trend obtained by the parameter variations.

### 4.1. Mapping and validation

In prior work (Dey and Dimitrov, 2022), we demonstrate that Loihi is capable of replicating the continuous dynamics of point neuronal models with high degree of precision and does so with much greater efficiency in terms of time and energy. The work comes with its challenges as simulations built on the conventional chips cannot be trivially mapped to the neuromorphic platform as its architecture differs remarkably from the conventional hardware. Classical simulations implemented on the Brain Modeling Toolkit (BMTK) serve as the foundation of our neuromorphic validation. To implement the mapping from one platform to the other, we introduce a re-scaling parameter *V*_*s*_ that transforms standard physical units used in BMTK to Loihi units and helps the mapping of the real-value representation of model states into Loihi's size-constrained integer-valued state representation, based on the available precision. In addition, we control the temporal state of the simulations through the voltage and current decay parameters which rely on the time-step *dt*. Thus, we build a function driven by two main arguments—membrane potential scale *V*_*s*_ and time-step *dt* that sets up the mapping protocol between the two platforms.

For validating the new simulations obtained by the above-mentioned mapping, we use both qualitative and quantitative measures. It can be seen that Loihi replicates BMTK very closely in terms of both membrane potential and current, the two state variables on which the Loihi LIF model evolves. The RMSE is found to be as low as 4.208 × 10^−5^ mV/ms with a correlation of ~0.9999 between the simulations. Furthermore, simulation results indicate Loihi is highly efficient in terms of speed and scalability as compared to BMTK, gaining acceleration up to a factor of 10^2^ (~10^3^ for larger networks).

Thus, this work demonstrates that classical simulations based on GLIF point neuronal models can be successfully replicated on Loihi with a reasonable degree of precision. Additionally, the high efficiency and low-power consumption of the neuromorphic platform with increasing network size paves the way for a complete replication of the mouse visual cortex dynamics, comprising hundreds of thousands of neurons and millions of synapses.

### 4.2. Perturbation and sensitivity analysis

Under this section, we examine how different state parameters of the LIF model impact the evolution of the state variables and ultimately affect our cost function. The goal here is to determine parameters that the model is more sensitive to and thus warrant a careful consideration as we map these values from one platform to the other.

This area deals with two central ideas—perturbation and sensitivity. Perturbation denotes the deviation of the parameters over a prescribed range of values from their initial point. On the other hand sensitivity highlights the effect of this perturbation on the model. We begin our work by establishing perturbation results that specifically target the underlying character of our cost function i.e., the ℓ_2_-norm. This analysis provides us a hint of the nature of the error curves as we perturb the parameters. Next, we perform a derivative-based analysis to establish the sensitivity of the model to the different parameter changes, i.e., higher the rate of change, the more its implication. From these mathematical analyses, we establish that the perturbation trend on our cost function would yield a global minimum, with the cost function increasing almost exponentially as we move away from the minimum. Moreover, the derivative-based analysis of our neural model helps establish the parameter that has the most significant impact on our model.

We verify our findings by enabling the simulations on both BMTK and Loihi. The groundwork established in the previous section sets a preamble for the execution of these implementations. However, we find that addressing the model differences becomes significant in establishing a comparable set of results. Thus, our sensitivity results offer a second set of validation for our previous validation results as the global minimum found here matches the RMSE value obtained earlier. Moreover, findings obtained from the mathematical analyses are supported by the simulations with the error curve following the predicted trend. Also, of the three parameters we choose to examine—membrane time constant (τ_*v*_), membrane capacitance (*C*_*m*_) (based on “*dt*”) and resting potential (*E*_*L*_) (based on “*V*_*s*_”), *E*_*L*_ impacts the model most significantly with an almost 42% increase in error in the first perturbation step as compared to 0.2% for the other two as was determined by the derivative based-analysis.

Thus, these results echo the importance of the model dynamics and the chosen norm function, and consequently our work sets a premise for further investigation on this topic based on more complex network and model dynamics.

### 4.3. Setting the foundation for future work

**Figure 8:**
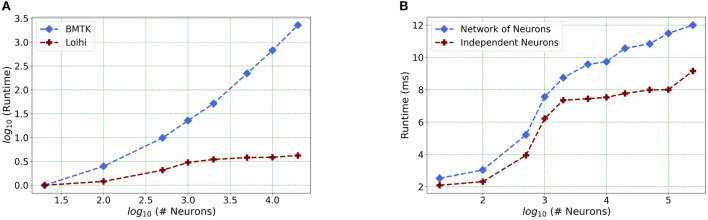
**(A)** Performance comparison between BMTK and Loihi for network sizes ranging from 1 to 20,000 for the simulation of 500 ms of dynamics. The values for each curve are scaled by the respective smallest runtime. The Loihi runtime units are in “*milliseconds*” and BMTK runtime is in “*seconds*.” **(B)** Loihi runtime for a network of up to 250K neurons for the simulation of 500 ms of dynamics.

Our future work is motivated by runtime performance comparisons for larger networks between the two platforms. As Loihi and BMTK are based on very different hardware systems that follow distinct dynamics and network-setup regimes, we use the runtime of the simulations to compare the performance of these implementations. As has been mentioned in the introduction, performance of Loihi far exceeds that of BMTK. Figure 8 compares the runtime of Loihi and BMTK, for running a network of randomly connected neurons with the same parameters. The network consists of excitatory and inhibitory neurons in a 1:1 ratio driven by bias current, with connection probability set at 0.1.

As can be seen from Figure 8A and Table 3, Loihi easily scales up to larger network sizes with a minuscule rise in runtime whereas for BMTK the increase is quite rapid. While both seem to exhibit a power-law scaling (string line on this graph), Loihi's scaling power is much smaller. It is also worth noting here that for Loihi the unit for the runtime are in “*milliseconds*” whereas for BMTK they are in “*seconds*.” Here we stop at 20,000 neurons as it can be inferred from the graph that increasing the network size would increase the time cost for BMTK substantially.

**Table 3:** Simulation runtime in Loihi and BMTK.

**Network size**	**Loihi time (ms)**	**BMTK time (s)**
20	2.52	0.12
100	3.03	0.3
500	5.21	1.13
1,000	7.56	2.72
5,000	9.57	26.47
10,000	9.73	80.45

Furthermore, following the above outcome, we extend our network size *in Loihi only* to 250K neurons in order to investigate what potential Loihi holds to execute the final goal of simulating about ~250,000 neurons with ~500 M synapses in the future, a simulation scale comprising much of the experimentally observed dynamics in the mouse visual cortex available to the AIBS. We record our observations for a randomly connected network of neurons as well as an independent set of unconnected neurons. From Figure 8B and Table 4, we can infer that the runtime remains consistent with the above result, with the independent set of neurons completing the simulation marginally faster. It is worth mentioning here that unconnected neurons will prove to be very useful during optimization (Schuman et al., 2020).

**Table 4:** Simulation runtime for a connected network and independent neurons in Loihi.

**Network size**	**Connected network (ms)**	**Independent neurons (ms)**
20	2.52	2.09
100	3.03	2.31
500	5.21	3.94
1,000	7.56	6.22
5,000	9.57	7.35
10,000	9.73	7.53
50,000	10.84	7.98
100,000	11.49	8.00
250,000	11.98	9.16

This shows that Loihi performs well for connected networks, setting the stage for our main aim for neural simulations. Additionally, it also works well for independent set of neurons which contribute to solutions of problems that require on-chip parameter and meta-parameter searches, e.g., for Evolutionary Programming (Schuman et al., 2020).

We do not asses the state-based cost for these networks as their large sizes require multi-chip simulations which we expect to be better supported on Loihi 2 (Intel, 2022). Furthermore, other research groups have firmly established that we cannot expect exact replication of subthreshold network states between simulators except for few very simple small networks (van Albada et al., 2018; Crook et al., 2020). Thus, on the network level we need to develop cost functions that capture appropriate network activity details on different scales (e.g., average spike rates and correlations on the coarsest levels, as in van Albada et al., 2018).

Thus, in closing for this section, we want to highlight that with the advent of Loihi 2 (Intel, 2022), we aim to address the limitations of the larger networks and carry out the next steps of our work in this new hardware. We are planning to investigate the full GLIF dynamics as we would have better support for more complex network topology and spiking dynamics. In addition, we hope to implement a connected network of 250K neurons with specific synaptic variables as available in the AIBS dataset. We also plan to investigate the control and performance of temporal precision choices. Till date, our limited conclusion for these cases is that ~ 1 ms timestep is sufficient. This need not generalize to networks in which other precision may be needed, with corresponding tradeoffs to changes in the parameters. We intend to explore this question further.

In terms of sensitivity analysis, investigation in the future would entail employing global sensitivity analysis methods to assess the impact of the parameters on a larger range. It would also be interesting to examine the impact of our results for more complex neural models, thus help bring in more insight about the intricacies of the model. Sensitivity analysis for different spike-based cost functions would be another avenue for exploration.

Thus, to sum up, we establish that Loihi is fully capable of reproducing biologically relevant neural networks and does it very efficiently. In spite of major architectural and model differences, Loihi is able to emulate the features presented by the conventional hardware simulations. This numerical validation, combined with the mathematical and computing knowledge of this new brain-like paradigm promises reliability for the ongoing expansion in the field of brain studies and neuroscience. The foundational work presented here equipped with the exploration of a novel neuromorphic regime furthers our exploration of true brain-scale networks and the various information processing principles of this very rich and complex organ—the brain.

## Data availability statement

The datasets presented in this study can be found in online repositories. The names of the repository/repositories and accession number(s) can be found at: https://github.com/srijanie03/bmtk_loihi_utils.

## Author contributions

SD and AD contributed to conception and design of the study. SD performed the simulations, analysis, and wrote the first draft of the manuscript. AD wrote parts of the manuscript, edited, and reviewed. All authors contributed to manuscript revision, read, and approved the submitted version.

## Footnotes

^1^This work was started when Loihi was first released. At present, Intel Corp. has released its second generation neuromorphic chip Loihi2 which is not used for this work. However, it is worth mentioning that the fundamentals of our analytical approach remain relevant for both chips.
